# Effect of clear aligners and fixed appliances among orthodontic patients: A comparative study

**DOI:** 10.6026/973206300213099

**Published:** 2025-09-30

**Authors:** Shashank Gaikwad, Mrunal Dave, Ananya Bhargava, Ishaan Saha, Shivani Kumari, Poonam Kumari Jayaprakash, Miral Mehta, Jugajyoti Pathi

**Affiliations:** 1 Department of Orthodontics and Dentofacial Orthopaedics, Bharati Vidyapeeth (Deemed to be University) Dental College and Hospital, Navi Mumbai, Maharashra, India; 2 Bethlehem Smile Design, Bethlehem, Pennsylvania, USA; 3 Consultant Orthodontist, Avanti Hospital, Ujjain, Madhya Pradesh, India; 4 Department of Dental Surgery, Sri Ramachandra Dental College & Hospital, SRIHER, Porur, Chennai, Tamil Nadu, India; 5 Department of Orthodontics and Dentofacial Orthopaedics, SGT University, Gurgaon-Badli Road Chandu, Budhera, Gurugram, Haryana, India; 6 Department of Orthodontics and Dentofacial Orthopaedics, Dental Institute, RIMS, Ranchi, Jharkhand, India; 7 Department of Pediatric and Preventive Dentistry, Karnavati School of Dentistry, Karnavati University, Gandhinagar, Gujarat, India; 8 Department of Oral & Maxillofacial Surgery, Kalinga Institute of Dental Sciences, KIIT Deemed to be University, Bhubaneswar, Odisha, India

**Keywords:** Clear aligners, fixed appliances, orthodontics, treatment efficacy, patient satisfaction, treatment duration, PAR index, comparative study

## Abstract

Orthodontic treatment can be delivered using clear aligners (CA) or fixed appliances (FA), yet direct comparisons of overall efficacy remain limited. This randomized controlled trial included 120 patients (60 CA, 60 FA) with mild-to-moderate malocclusions to evaluate treatment duration, occlusal outcomes (PAR index), pain, satisfaction and compliance. Treatment duration was longer with CA (18.2 ± 3.1 vs. 14.5 ± 2.8 months; p<0.001), while both groups achieved comparable PAR index reductions (p=0.082). Pain was lower and satisfaction higher in the CA group, with good compliance (21.8 ± 1.5 h/day). Both modalities effectively corrected malocclusions, with FA showing greater efficiency and CA providing superior comfort and patient experience.

## Background:

Malocclusion, a deviation from ideal occlusion, is a prevalent condition affecting individuals worldwide, with potential impacts on oral function, aesthetics and psychosocial well-being [1]. Orthodontic treatment plays a crucial role in correcting these discrepancies, utilizing various appliances to move teeth through controlled application of biomechanical forces [2]. Traditionally, fixed orthodontic appliances (FA), comprising brackets bonded to teeth and archwires have been the gold standard for treating a wide spectrum of malocclusion severities due to their precise control and predictability [3]. In recent decades, clear aligner therapy (CA) has emerged as a popular alternative, particularly among adult patients seeking a more aesthetic and removable treatment option [4]. CA utilizes a series of custom-made, transparent polyurethane trays that apply incremental forces to move teeth sequentially [5]. Proponents highlight advantages such as improved aesthetics, enhanced oral hygiene access, greater comfort and the ability to remove appliances during eating and special occasions [6]. However, concerns persist regarding their limitations in managing complex tooth movements (e.g., significant extrusion, rotation correction, root torque) and potential dependence on patient compliance [7]. The efficacy of orthodontic treatment is multifaceted, encompassing not only the achievement of ideal occlusion and stability but also treatment efficiency, patient experience and long-term outcomes [8]. While numerous studies have documented the effectiveness of both FA and CA individually [9, 10], direct comparative research evaluating comprehensive efficacy metrics across comparable patient populations remains relatively limited, especially concerning contemporary aligner systems and diverse malocclusion types [11]. Recent systematic reviews suggest comparable occlusal outcomes for mild-to-moderate cases but often report longer treatment durations with aligners [12, 13]. However, significant heterogeneity exists in study designs, sample characteristics, outcome measures, and the specific aligner systems evaluated, making definitive conclusions challenging. Furthermore, robust data comparing patient-reported outcomes like pain and satisfaction between these modalities in a controlled setting are scarce [14]. A critical research gap exists in the prospective, randomized comparison of modern CA systems with conventional FA using standardized, objective outcome measures (like the Peer Assessment Rating - PAR index) alongside validated patient-reported outcome measures (PROMs) within a well-defined patient cohort. Understanding the relative strengths and weaknesses of each approach across key efficacy dimensions is essential for evidence-based clinical decision-making and personalized treatment planning [15]. Therefore, it is of interest to conduct a prospective, randomized controlled trial to comprehensively evaluate and compare the efficacy of Clear Aligners and fixed appliances in orthodontic patients.

## Materials and Methods:

## Study design and setting:

This prospective, parallel-group, randomized controlled trial (RCT) was conducted in the Department of Orthodontics at a tertiary care dental hospital between January 2020 and December 2022.

## Sample size calculation:

The sample size was calculated based on the primary outcome variable of treatment duration. Assuming a clinically significant difference of 2.5 months between groups (standard deviation of 3.0 months), an alpha level of 0.05, and a power (1-β) of 90%, a minimum sample size of 52 participants per group was required. Accounting for a potential 15% dropout rate, the final target sample size was set at 60 participants per group (total N=120).

## Participants:

Inclusion Criteria: Patients aged 18-35 years; non-syndromic; presenting with mild-to-moderate malocclusion (Angle Class I or Class II division 1, requiring non-extraction treatment or extraction of maximum two premolars per quadrant); requiring comprehensive orthodontic treatment; good general and oral health; adequate periodontal support (probing depth ≤ 3mm, no bleeding on probing); committed to regular appointments; able to understand and provide informed consent. Exclusion Criteria: Severe malocclusion (e.g., Class III, skeletal discrepancies requiring orthognathic surgery, severe crowding >8mm); active periodontal disease; extensive restorative needs; craniofacial anomalies; history of temporomandibular joint disorders; pregnancy or lactation; previous orthodontic treatment; inability to comply with study procedures or aligner wear requirements.

## Randomization and blinding:

Eligible participants were randomly allocated to either the Clear Aligner (CA) group or the Fixed Appliance (FA) group using a computer-generated block randomization sequence (block size of 4) prepared by an independent statistician. Allocation concealment was maintained using sequentially numbered, opaque, sealed envelopes opened by the treating orthodontist only after baseline assessment and consent. Due to the inherent nature of the interventions, blinding of participants and treating clinicians was not feasible. However, the outcome assessors (calibrated orthodontists not involved in treatment) and the statistician analyzing the data were blinded to group allocation.

## Interventions:

Clear Aligner Group (CA): Participants received treatment using a commercially available clear aligner system (Invisalign Full, Align Technology, San Jose, CA, USA). Digital impressions (iTero Element scanner, Align Technology) were taken, and treatment plans were developed using proprietary software (ClinCheck). The plan was reviewed and approved by the treating orthodontist before aligner fabrication. Participants were instructed to wear each aligner for 22 hours per day, changing to the next set every 1-2 weeks as per the ClinCheck schedule. Attachments, interproximal reduction (IPR), and auxiliaries (e.g., Class II elastics, buttons) were used as clinically indicated. Compliance was monitored subjectively at each visit and objectively via the aligner's built-in wear-time indicator (if available) or patient logs. Fixed Appliance Group (FA): Participants received treatment with conventional pre-adjusted edgewise fixed appliances (0.022" slot, MBT prescription, 3M Unitek, Monrovia, CA, USA). Treatment involved bonding of metal brackets to all fully erupted teeth. Initial alignment was achieved using nickel-titanium archwires, followed by stainless steel archwires for space closure, detailing, and finishing. Auxiliaries such as elastomeric chains, coil springs, and intermaxillary elastics were used as needed. Standard oral hygiene instructions were provided, including the use of interdental brushes and floss threaders.

## Outcome measures:

## Primary outcome:

Treatment Duration: Defined as the time elapsed (in months) from the date of appliance placement (bonding for FA, delivery of first aligner for CA) to the date of appliance removal/debonding and delivery of retainers.

## Secondary outcomes:

Occlusal Outcome: Assessed using the Peer Assessment Rating (PAR) index. Pre-treatment (T0) and post-treatment (T1) dental casts were digitally scanned (3Shape E4 scanner, Copenhagen, Denmark) and scored by two calibrated, blinded examiners (intraclass correlation coefficient >0.90). The percentage reduction in PAR score was calculated: [(T0 PAR - T1 PAR) / T0 PAR] * 100%. Pain Perception: Measured using a 100-mm Visual Analog Scale (VAS), where 0 = "No pain" and 100 = "Worst imaginable pain". Participants recorded peak pain levels experienced during the first 24 hours after appliance placement (FA bonding/CA first aligner) and after each subsequent archwire change (FA) or aligner change (CA). The mean peak pain score across all recorded time points was calculated for each participant. Patient Satisfaction: Assessed at treatment completion (T1) using a validated 5-point Likert scale questionnaire (1=Very Dissatisfied, 5=Very Satisfied) covering aspects of aesthetics, comfort, convenience, function, and overall treatment experience. A mean overall satisfaction score was calculated. Compliance (CA Group Only): Mean daily aligner wear time (hours/day) was estimated based on patient self-report logs and, where available, objective data from aligner indicators during active treatment phases. Treatment Complications: Incidence of minor complications requiring unscheduled visits was recorded, including broken attachments/loose brackets, lost aligners/brackets, broken archwires, and ulcerations requiring intervention.

## Data collection:

Baseline data (demographics, malocclusion type, T0 PAR score) were collected at enrolment. Pain scores were recorded by participants at home after specified appointments and collected at the next scheduled visit. Compliance logs (CA group) were reviewed monthly. T1 PAR scores, satisfaction questionnaires, and treatment duration were recorded at treatment completion. Complication data were documented prospectively throughout treatment.

## Statistical analysis:

Data were analyzed using SPSS software (version 27.0, IBM Corp., Armonk, NY, USA). Normality of distribution was assessed using the Shapiro-Wilk test. Continuous variables (treatment duration, PAR reduction %, pain VAS, satisfaction score, compliance hours) were presented as mean ± standard deviation (SD) and compared between groups using the independent samples t-test. Categorical variables (gender, malocclusion type, complication incidence) were presented as frequencies (percentages) and compared using the chi-square test or Fisher's exact test as appropriate. A p-value < 0.05 was considered statistically significant. Intention-to-treat analysis was employed, with missing data handled using multiple imputation.

## Results:

A total of 156 patients were screened for eligibility. Twenty-six patients were excluded (18 did not meet the inclusion criteria, 8 declined participation). One hundred twenty participants were randomized (60 CA, 60 FA). Five participants in the CA group and three in the FA group were lost to follow-up or withdrew consent. Data from the remaining 112 participants (55 CA, 57 FA) were included in the final analysis (Table 1 - see PDF). The mean treatment duration was significantly longer in the Clear Aligner group (18.2 ± 3.1 months) compared to the Fixed Appliance group (14.5 ± 2.8 months; p<0.001). Both groups achieved substantial and statistically significant improvements in occlusion, as measured by the PAR index reduction percentage. The CA group showed a mean reduction of 78.4 ± 8.2%, while the FA group showed a mean reduction of 82.1 ± 7.5%. Although the FA group demonstrated a numerically higher reduction, this difference did not reach statistical significance (p=0.082) (Table 2 - see PDF). Patient-reported outcomes revealed significant differences. The mean peak pain score (VAS) was significantly lower in the CA group (3.8 ± 1.2) compared to the FA group (6.2 ± 1.5; p<0.001). Conversely, patient satisfaction at treatment completion was significantly higher in the CA group (4.5 ± 0.6) than in the FA group (3.8 ± 0.7; p<0.001). Compliance in the CA group, as estimated by self-report and indicators, averaged 21.8 ± 1.5 hours per day (Table 2 - see PDF). The overall incidence of minor complications requiring unscheduled visits was 34.5% in the CA group and 40.4% in the FA group, with no statistically significant difference (p=0.421). The most common complications in the CA group were broken attachments (21.8%) and lost aligners (12.7%), while loose brackets (31.6%) were most frequent in the FA group. The incidence of soft tissue ulcerations was comparable between groups (CA: 9.1%, FA: 14.0%; p=0.384) (Table 3 - see PDF).

## Discussion:

This prospective randomized controlled trial provides a comprehensive comparison of the efficacy of Clear Aligners and fixed appliances in treating mild-to-moderate malocclusions in young adults. The findings highlight distinct profiles for each modality across key efficacy dimensions: treatment efficiency, occlusal outcomes, patient experience, and complication rates. The significantly longer treatment duration observed in the Clear Aligner group aligns with several previous studies and systematic reviews [16, 17]. This difference can be attributed to several factors inherent to aligner therapy. The sequential nature of tooth movement with aligners inherently extends the time required for comprehensive tooth movement compared to the continuous forces applied by fixed appliances [18]. Furthermore, the reliance on patient compliance for adequate wear time introduces variability; even minor lapses can accumulate and prolong treatment. While the mean compliance in our CA group was high, achieving perfect consistency over 18+ months is challenging. Additionally, the need for mid-course corrections, refinements, or attachments to achieve complex movements can add significant time, a factor less frequently required with FA once the mechanics are established [19]. The efficiency of FA in closing extraction spaces and performing precise root movements remains a key advantage [20]. Despite the difference in duration, both modalities achieved substantial and clinically meaningful improvements in occlusion, as evidenced by high PAR index reduction percentages. The lack of a statistically significant difference in PAR reduction (p=0.082) suggests that, for the mild-to-moderate cases studied, both systems are capable of delivering excellent occlusal outcomes when treatment is completed. This finding is consistent with recent RCTs and meta-analyses focusing on non-extraction and mild extraction cases [21, 22]. The numerical trend towards slightly higher PAR reduction with FA might reflect their superior control over certain tooth movements, particularly root torque and rotation, which are critical for optimal finishing and stability [23]. However, the clinical significance of this small difference (3.7%) in the context of overall success is debatable, especially considering the advantages CA offers in other domains. The most pronounced differences favouring clear aligners were observed in patient-reported outcomes. Significantly lower peak pain scores in the CA group corroborate findings from previous studies [24, 25]. The removability of aligners allows patients to take breaks during periods of peak discomfort (e.g., after changing aligners), and the thermoplastic material exerts lighter, more distributed forces compared to the initial forces from nickel-titanium archwires engaging misaligned teeth in FA [26]. This reduced pain perception likely contributes significantly to the higher patient satisfaction scores observed in the CA group. The aesthetic advantage of aligners is a well-documented driver of satisfaction, particularly among adults [27]. Furthermore, the ease of oral hygiene maintenance and the ability to eat without dietary restrictions enhance the overall treatment experience for CA users [28]. While FA patients generally report satisfaction with their final results, the higher burden of discomfort, dietary limitations, and challenges with oral hygiene during active treatment likely contributed to their lower satisfaction scores in this study. The overall incidence of minor complications was similar between groups. This finding suggests that while the types of complications differ (attachments/lost aligners vs loose brackets/broken wires), the overall burden of unscheduled visits related to appliance issues is comparable. The high rate of loose brackets in the FA group is consistent with clinical experience and literature [29]. Broken attachments and lost aligners were the primary issues in the CA group, emphasizing the importance of patient education and robust attachment bonding protocols. The comparable ulceration rates indicate that both appliances can cause soft tissue irritation, though the causes differ (aligner edges/attachments vs bracket hooks/archwire ends). Effective patient education on managing these issues is crucial for both modalities.

## Conclusion:

Both Clear Aligners and fixed appliances are effective treatment options for mild-to-moderate malocclusions. While fixed appliances provide greater treatment efficiency, Clear Aligners enhance patient comfort and satisfaction. The choice of modality should be guided by clinical requirements and individual patient preferences.

## References

[1] Alam MK (2024). J Pharm Bioallied Sci..

[2] Alfawzan AA (2024). J Pharm Bioallied Sci..

[3] Annamalaisamy S (2024). J Pharm Bioallied Sci..

[4] Martin C (2023). Cochrane Database Syst Rev..

[5] Liu F (2024). BMC Oral Health..

[6] Jaber ST (2022). Cureus..

[7] Ke Y (2019). BMC Oral Health..

[8] Alam MK (2024). J Pharm Bioallied Sci..

[9] Al-Gumaei WS (2025). Clin Oral Investig..

[10] Chattopadhyay J (2024). Bioinformation..

[11] Paglia L, Marzo G (2023). Eur J Paediatr Dent..

[12] Baneshi M (2025). J Evid Based Dent Pract..

[13] Jyotirmay et (2021). J Contemp Dent Pract..

[14] Jaber ST (2023). Cureus..

[15] Irsheid R (2024). Clin Oral Investig..

[16] Brown M (2022). Am J Orthod Dentofacial Orthop..

[17] Kotsailidi EA (2025). Clin Adv Periodontics..

[18] Li Y (2020). Prog Orthod..

[19] Aref S (2024). J Pharm Bioallied Sci..

[20] Malpartida-Pacheco MI, Dulanto-Vargas JA. (2023). Rev Cient Odontol (Lima)..

[21] Ertugrul BY, Veli I (2022). J Stomatol Oral Maxillofac Surg..

[22] Chandra A (2024). Cureus..

[23] Papageorgiou SN (2020). Eur J Orthod..

[24] Kwon T (2023). Orthod Craniofac Res..

[25] Zheng J (2024). Front Cell Infect Microbiol..

[26] Menéndez López-Mateos  C (2022). J Clin Med..

[27] Vigneshkumar V (2024). J Pharm Bioallied Sci..

[28] Sinha A (2024). J Pharm Bioallied Sci..

[29] Grünheid T (2022). Am J Orthod Dentofacial Orthop..

